# Genome Sequence of Enterococcus faecium Strain ICIS 96 Demonstrating Intermicrobial Antagonism Associated with Bacteriocin Production

**DOI:** 10.1128/genomeA.00126-18

**Published:** 2018-03-08

**Authors:** Tatiana M. Pashkova, Alexey S. Vasilchenko, Yuriy A. Khlopko, Elena E. Kochkina, Olga L. Kartashova, Maria V. Sycheva

**Affiliations:** a Institute of Cellular and Intracellular Symbiosis, RAS, Orenburg, Russian Federation; b Orenburg State Agrarian University, Orenburg, Russian Federation; c Tyumen State University, Tyumen, Russian Federation

## Abstract

We report here the complete genome sequence of Enterococcus faecium strain ICIS 96, which was isolated from the feces of a horse. Bacteriological characterization of strain ICIS 96 revealed the absence of pathogenicity factors, while its spectrum of antagonistic activity was found to be broad, having activities associated with both Gram-positive and Gram-negative bacteria. Analysis of the E. faecium ICIS 96 genome revealed five genes associated with antimicrobial activity (enterocin [ent] A, ent B, lactobin A/cerein 7b, and ent L50 A/B). No genes that correlate with human pathogenicity were identified.

## GENOME ANNOUNCEMENT

Enterococci are among the most important bacteria found within the intestinal microbiota of humans and animals (1). The significance of enterococci is controversial, since these bacteria can cause infection and disease while at the same time they are actively used in the food industry. Enterococcus faecium isolates from animals are not particularly dangerous to humans, but they can transmit antimicrobial resistance genes for other pathogenic enterococci (2). An important benefit of enterococci is their ability to produce bacteriocins (3).

E. faecium strain ICIS 96 was isolated from the feces of a healthy horse using bacteriological approaches. E. faecium ICIS 96 demonstrated broad antagonistic activity against Listeria spp., Escherichia coli K-12, and vancomycin-resistant clinical isolates of E. faecalis. In addition, ICIS 96 is characterized by the absence of any phenotypic manifestation of pathogenicity factors, namely, DNase, gelatinase, and hemolytic activity. Preparation of DNA libraries and sequencing were carried out at the Institute for Cellular and Intracellular Symbiosis UrB RAS (Orenburg, Russia). Sequencing was performed on an Illumina MiSeq platform using a MiSeq version 3 reagent kit (Illumina, San Diego, CA, USA).

Genomic DNA was extracted from an overnight culture of E. faecium ICIS 96 and used to prepare a DNA library with the Nextera XT DNA sample preparation kit (Illumina). The reads were quality trimmed using the sliding window mode of the Trimmomatic program (4). *De novo* genome assembly was performed using the SPAdes genome assembler (St. Petersburg genome assembler, version 3.7.1) (5).

The assembly yielded 364 contigs composed of a total of 2,748,236 bp, with *N*_50_ and *L*_50_ sizes of 16,483 bp and 55 bp, respectively. Strain ICIS 96 has a G+C content of 37.97% and an average coverage of 17.0×. The genome sequence was annotated using the NCBI Prokaryotic Genome Annotation Pipeline (PGAP) (https://www.ncbi.nlm.nih.gov/genome/annotation_prok), which revealed 2.947 coding sequences, including 2,689 proteins, 181 pseudogenes, 11 rRNAs (5 5S, 3 16S, and 3 23S), 62 tRNAs, and 4 noncoding RNAs.

Analysis of the E. faecium ICIS 96 genome revealed genes involved in resistance to some antimicrobials such as copper, glycopeptide (bleomycin), and ionophore antibiotic (tetronasin). The bacteriocin-producing potential of E. faecium ICIS 96 is determined by the presence of five bacteriocin-related genes (ent A, ent B, lactobin A/cerein 7b, and ent L50 A/B). No genes that correlate with human pathogenicity were identified with the Rapid Annotations using Subsystems Technology (RAST) server (6).

The genetic features of this strain are of particular interest, and E. faecium ICIS 96 can serve as a model strain for studies investigating bacterial antagonism associated with the production of bacteriocins. In addition, the revealed properties of E. faecium ICIS 96 may be crucial for further probiotic development and biopreservation.

### Accession number(s).

This whole-genome shotgun project has been deposited in DDBJ/ENA/GenBank under the accession number NNSQ00000000.
